# Strollur: an R package for working with amplicon sequence data in R

**DOI:** 10.1128/mra.00839-26

**Published:** 2026-09-29

**Authors:** Sarah L. Westcott, Gregory Johnson, Patrick D. Schloss

**Affiliations:** 1 Department of Microbiology & Immunology, University of Michigan 1259, Ann Arbor, Michigan, USA; University of Strathclyde, Glasgow, United Kingdom

**Keywords:** microbiome, bioinformatics, R, amplicon sequence data, 16S rRNA gene sequences

## Abstract

Microbiologists are increasingly relying on R to analyze and visualize amplicon sequence data from microbiome studies. We present strollur, which is an open-source package that facilitates the import, export, and handling of amplicon sequence data within the R programming language.

## ANNOUNCEMENT

Amplicon sequencing projects, including 16S rRNA gene sequence collections, are widely used by microbiologists to analyze diverse microbiomes. These data sets are often large and complicated, making it difficult for researchers to keep their data organized and record the provenance of derivative data products. A long-standing difficulty for many has been the ability to move data between different analytical tools. This often requires reformatting data, which is challenging for researchers with limited programming skills. Several efforts have been made to facilitate the management of amplicon sequencing data and their associated metadata, including the biom file format (1), mothur output files (2), the qiime2 archive (3), and phyloseq R objects (4). Each of these formats and tools has strengths but also limitations, which make their use difficult for researchers new to microbiome data analysis or who are more accustomed to using R’s tidyverse collection of packages.

To overcome these challenges, we developed the strollur R package. Our goal was to develop a framework to store amplicon sequence data that could be integrated within other packages without the end user necessarily knowing they were using strollur. This is meant to be analogous to how many users of the tidyverse use the tibble package without knowing they are using the package. By analogy, strollur strives to provide a consistent interface to amplicon sequence data, which also integrates with analysis and visualization tools from the tidyverse and other tools used by microbiome researchers.

**Fig 1:**
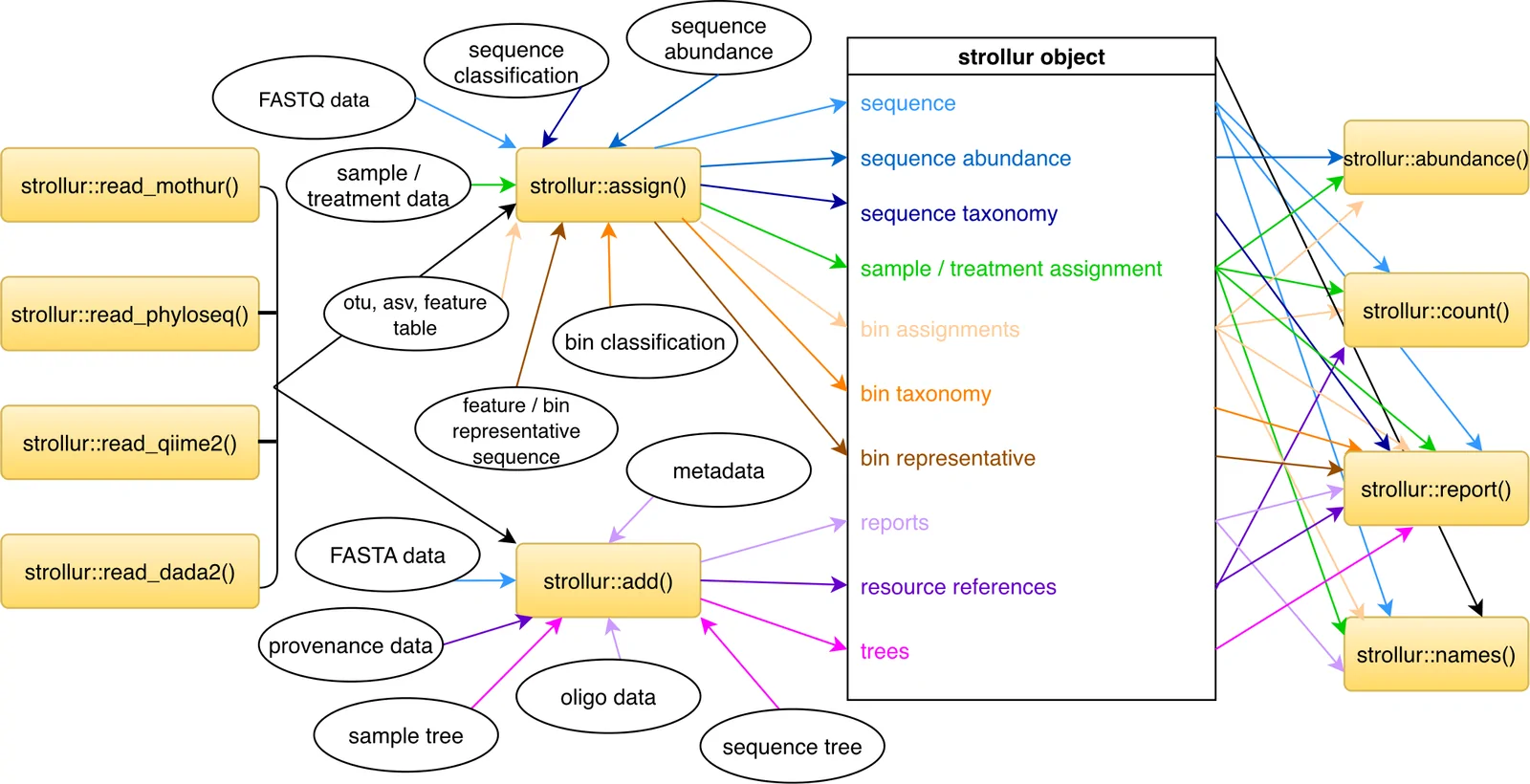
The strollur R package facilitates the import of microbiome data generated by popular software packages into R and allows users to export the data in a variety of formats.

Strollur’s data structures allow users to keep track of sequence data, abundance data, classification data, phylogenetic trees, operational taxonomic unit (OTU), and amplicon sequence variant assignments, reports from different processing steps, and sample metadata (Fig. 1). Furthermore, the data structures can accommodate information describing the commands that generated the data products and information about the databases that were used. The package includes user-facing functions for getting, assigning, and summarizing various forms of data. The package also provides functions to read data from or write data to other popular formats, including mothur (2), QIIME 2 (3), DADA2 (5), phyloseq (4), and BIOM (1). Strollur can be used on any operating system with no additional dependencies.

Strollur also includes functions designed for developers to integrate strollur objects into their own R packages. These developer-facing functions provide additional access to the back-end data. The ability to import and export data as a strollur object has already been integrated into the clustur (6) and phylotypr (7) packages. The package leverages the R6 object-oriented programming framework and the Rcpp package to accelerate data processing. By using these features of R, strollur minimizes the need to create deep copies of large data objects.

## Data Availability

Strollur can be accessed through the Comprehensive R Archive Network (CRAN; DOI: 10.32614/CRAN.package.strollur) and developmental versions are available through the project’s GitHub website (https://github.com/mothur/strollur). The package’s GitHub repository includes the package’s source code, the website’s source code, automated tests written using the testthat R package, and an issue tracker where users can post questions, bug reports, and suggestions for future features. A pkgdown version of the documentation including multiple articles describing its use is hosted at (https://mothur.org/strollur). The strollur package is available under the GNU General Public License (v3).
